# Influence of preprandial vs. postprandial insulin glulisine on weight and glycaemic control in patients initiating basal-bolus regimen for type 2 diabetes: a multicenter, randomized, parallel, open-label study (NCT00135096)

**DOI:** 10.1111/j.1463-1326.2011.01478.x

**Published:** 2011-12

**Authors:** R Ratner, A Wynne, S Nakhle, O Brusco, A Vlajnic, M Rendell

**Affiliations:** 1 MedStar Health Research InstituteHyattsville, MD, USA; 2 Cotton O'Neil ClinicTopeka, KS, USA; 3 VA Health Care SystemLas Vegas, NV, USA; 4 Private PracticeCorpus Christi, TX, USA; 5 Sanofi-Aventis U.S.Bridgewater, NJ, USA; 6 Diabetes Center at Creighton UniversityOmaha, NE, USA

**Keywords:** glycaemic control, insulin, insulin glargine, insulin glulisine, postprandial

## Abstract

**Aim:** Insulin therapy is commonly associated with weight gain. The timing of prandial insulin administration may enhance its efficacy/safety and maintain effective weight control. This study examined the effect of postprandial vs. preprandial insulin glulisine on weight gain and glycaemic control in type 2 diabetes patients taking basal insulin.

**Methods:** This was a multicenter, randomized, open-label trial conducted in 45 centres in the USA. A total of 716 patients with type 2 diabetes and glycated haemoglobin A_1c_ (HbA_1c_) ≥7.5% and ≤10.0% were screened; 345 were randomized and 322 comprised the intent-to-treat group (premeal, 163; postmeal, 159). Insulin glargine once daily, ±metformin and subcutaneous injections of premeal or postmeal insulin glulisine were given for 52 weeks. Main outcome measures included changes in HbA_1c_, fasting plasma glucose and weight from study baseline to endpoint (week 52).

**Results:** At study end, insulin glulisine achieved similar glycaemic control whether it was administered before or after meals (HbA_1c_: 7.04% premeal vs. 7.16% postmeal, p = NS). Overall hypoglycaemia incidence and severe hypoglycaemia rates were not significantly different between premeal and postmeal groups; however, symptomatic and nocturnal hypoglycaemia rates were higher in the postprandial group. Mean body weight was lower in the postmeal group, with the difference between postmeal and premeal weight change from baseline to week 52 of −0.87 kg (p = 0.243).

**Conclusion:** Postprandial glulisine administration provided similar glycaemic control and was non-inferior to preprandial administration on weight gain, without additional risk of severe hypoglycaemia, showing dosing flexibility and the feasibility of such approach when clinically indicated.

## Introduction

The prevalence of type 2 diabetes continues to increase, as does the number of obese individuals in the USA, with 80 to 90% of patients with type 2 diabetes being overweight or obese [1]. It is widely recognized in published literature [2–4], as well as in consensus treatment guidelines [5], that insulin is highly effective in reducing blood glucose (BG) and maintaining glycaemic control. Type 2 diabetes is a progressive disease characterized by ongoing loss of *β*-cell function leading to oral agent failure and the need for insulin-replacement therapy. Treatment with basal insulin generally provides effective glycaemic control for most patients. Over time, however, the addition of prandial insulin to basal therapy is often required for patients to minimize postprandial glucose excursions and maintain glycaemic control. The combination of basal and prandial insulin therapy closely mimics the normal physiologic pattern of endogenous insulin release and improves glycaemic control [3,6]. Intensive, physiologic insulin replacement in this manner, however, has been associated with undesired weight gain [7].

Recent years have witnessed the development of several novel antihyperglycaemic agents, as well as advances in the delivery and pharmacology of insulin therapy to help patients meet their glycaemic goals [8]. The currently available options for prandial insulin therapy include regular human insulin (RHI) and rapid-acting insulin (RAI) analogues. RAIs have several advantages over RHI, including a time-action profile that more closely mimics endogenous insulin, the capability of injection within 15 min of a meal compared with 30–45 min required for RHI, and better control of postprandial glucose excursions [9]. Because of changing lifestyles, including variable meal times, adherence to the 30-min injection-premeal lag time typically recommended for RHI can be difficult for many patients [10]. Therefore, it is important to identify treatments that can help patients achieve their glycaemic goals while providing effective flexible insulin dosing options.

Insulin glulisine is an RAI analogue that displays a more rapid onset, an earlier peak effect, and a shorter duration of action than RHI [3,11], with uniform absorption and efficacy among wide ranges of body mass indexes (BMIs) [12]. The time-action profile of insulin glulisine may provide the advantage of adjusting doses according to actual food intake, as this insulin is also indicated for use within 20 min after the start of a meal [13]. Dose adjustment, in turn, may allow patients to decrease the meal size, thereby enhancing the effect of insulin in improving glycaemic control and modulating body weight.

Postmeal administration of insulin glulisine has been studied in patients with type 1 diabetes, and was shown to provide glycaemic control equivalent to that of premeal insulin glulisine and RHI, with a small but statistically significant body weight loss over 12 weeks [6]. In addition, no differences in symptomatic or nocturnal hypoglycaemia rates were shown between groups [6]. These results have not yet been confirmed in patients with type 2 diabetes.

This study was undertaken to assess whether postprandial use of insulin glulisine would be non-inferior to preprandial use in glycaemic control with particular attention to weight changes in patients with type 2 diabetes suboptimally controlled with oral antihyperglycaemic agents and insulin.

## Methods

### Patients

Three hundred and forty-five patients were recruited from 45 centres in the USA. Patients were male and female, aged 18–70 years, with a diagnosis of type 2 diabetes for at least 6 months, and with inadequately controlled diabetes (HbA_1c_≥7.5% and ≤10.0% at screening) on current therapy. Patients were required to have been on insulin therapy with at least two injections per day ±metformin for a minimum of 3 months prior to the study entry. All participants showed the ability and willingness to perform self-monitoring of blood glucose (SMBG) at least four times per day and at least seven times daily during the seven-point BG profile measurement days.

Patients were excluded from this study if they were treated with sulfonylureas, thiazolidinediones, or any other oral antidiabetic drugs other than metformin within 3 months prior to the study entry; were treated with metformin and had serum creatinine levels of ≥132.6 µmol/l (male patients) or ≥123.8 µmol/l (female patients); or hepatic disease; had serum alanine aminotransferase or aspartate aminotransferase levels >2.5 times the upper limit of normal.

### Study Design

This was a multicenter, controlled, open-label, 1 : 1, randomized, parallel-group study consisting of a 2-week screening period and a 52-week treatment period. During screening, qualified patients remained on their pre-existing insulin regimen and received dietary and lifestyle recommendations from a health care professional. Patients then were randomized to: (i) premeal arm—patients received insulin glulisine three times daily, 0–15 min before the three main meals, and insulin glargine once daily, ±metformin or (ii) postmeal arm—patients received insulin glulisine three times daily, 20 min after the start of a meal, and insulin glargine once daily, ±metformin.

### Treatments

All patients received a single daily subcutaneous (SC) injection of insulin glargine, the initial dose being 50% of the total daily insulin dose from the patient's previous treatment. The timing of insulin glargine administration was based on the needs of individual patients. A change in timing was permitted during the first 6 months after randomization if deemed appropriate by the investigator; however, the change could be made only once per patient. Weekly dose titration was based on the mean of the fasting 3-day SMBG values to achieve a fasting plasma glucose (FPG) level of <6.1 mmol/l (110 mg/dl) and an HbA_1c_ level of <7.0% (Table 1). The dose increase could be split into two or more incremental increases over the course of the week.

**Table 1:** Insulin glargine titration algorithm

HbA_1c_	Mean fasting 3-day SMBG (mmol/l)	Insulin glargine adjustment (U)
≥7.0%	>10.0	Increase 8 U
	7.8–10.0	Increase 6 U
	6.7–7.7	Increase 4 U
	6.1–6.6	Increase 2 U
	5.6–6.0	Increase 0–2 U at investigator's discretion*
	3.9–5.5	No change
	<3.9	Decrease the dose of insulin glargine by 10%
<7.0%	<3.9	Decrease the dose of insulin glargine by 10%

HbA_1c_, glycated hemoglobin A_1c_; SMBG, self-monitored blood glucose.

* If the HbA_1c_ was ≥7.0% and the mean FPG was between 5.6 and 6.0 mmol/l (100 and 109 mg/dl), the investigator decided if the patient should receive a dose increase or not.

Insulin glulisine was administered SC, either 0–15 min before each main meal or 20 min after the start of a meal in accordance with product labelling. The initial dose of insulin glulisine in both arms was 50% of the total daily insulin dose from the patient's previous treatment. The total bolus insulin was divided to provide appropriate mealtime coverage; three sixths of the daily bolus dose was given with the largest meal (∼50%), two sixths—with the second largest meal (∼33%), and one sixth—with the smallest meal (∼17%). Patients randomized to the postmeal arm received insulin glulisine 20 min after the start of each meal, and the dose was adjusted based on the actual food intake during that specific meal. For example, if a full planned meal was consumed, 100% of the planned dose was taken; if half of the planned meal was consumed, 50% of the planned dose was taken; and if one fourth of the planned meal was consumed, 25% of the planned dose was taken. Weekly dose titration for adjusting premeal and postmeal insulin glulisine was based on a pattern management approach of preprandial SMBG values previously described by Bergenstal and coworkers [14]. Briefly, insulin glulisine dose adjustment for both treatment groups was based on specific patterns of prelunch, predinner and prebedtime (collectively referred to as mealtime) prandial BG levels, with targets for preprandial (before lunch and dinner) BG of <6.1 mmol/l (110 mg/dl) and bedtime BG of <7.2 mmol/l (130 mg/dl, Table 2). Patients receiving metformin at the time of randomization continued receiving the same dose throughout the entire treatment phase.

**Table 2:** Insulin glulisine titration algorithm

Mealtime dose	Pattern of low preprandial blood glucose values*	Pattern of high preprandial blood glucose values†
≤10 U	Decrease by 1 U	Increase by 1 U
11–19 U	Decrease by 2 U	Increase by 2 U
≥20 U	Decrease by 3 U	Increase by 3 U

* Two or more values of <3.9 mmol/l (70 mg/dl).

† Four or more values of ≥6.1 mmol/l (110 mg/dl) or 4 or more values of ≥7.2 mmol/l (130 mg/dl) at bedtime.

### Study Outcomes

The primary efficacy objective was to compare weight change from baseline to week 52 in patients receiving postmeal vs. premeal insulin glulisine with insulin glargine as basal insulin. Additional efficacy assessments included a comparison of total insulin dose; change in weight from baseline to weeks 3, 6, 12, 20, 28, 36, 44 and 52; proportion of patients experiencing >5% and >10% change in body weight; HbA_1c_ measurements at weeks 0, 12, 28, 36 and 52, or at study discontinuation between patients receiving insulin glulisine before meals and after meals; and proportion of patients achieving HbA_1c_ <7.0% and <6.5%. Safety endpoints included the incidence and rate of hypoglycaemic events, based on severity, recorded by each patient in the patient diary.

### Statistical Analysis

Data analyses were conducted based on three specified populations. The per-protocol (PP) population (the main population for the non-inferiority testing of the primary endpoint) included all randomized patients who took study medication, had no major protocol violations, and had body weight recorded at baseline and week 52. The intent-to-treat (ITT) population consisted of randomized patients with baseline measurements and at least one postbaseline measurement of weight and HbA_1c_ taken after randomization and was the main population for the superiority testing of the secondary endpoints. This population excluded subjects from 1 study site for Good Clinical Practice non-compliance. The safety analysis population consisted of all patients who took at least one dose of study medication.

Comparison of treatment effect on body weight change was based on a non-inferiority hypothesis with an upper bound from a two-tailed 95% confidence interval (CI) on the difference of the mean weight change of ≤1.5 kg between the two treatment arms. At each study site, the same scale was used at every visit for weight, without coats, sweaters, shoes, and with empty pockets. The non-inferiority in the change from baseline to week 52 in weight was investigated using analysis of covariance (ANCOVA; PROC MIXED in SAS). The model included change from baseline as a dependent variable; treatment, pooled site, and randomization strata (including the number of daily injections prior to the study, use of metformin at randomization, and injection methodology) as fixed effects; and baseline weight as a covariate.

The change from baseline to study time points in weight, HbA_1c_, FPG, dose of insulin glargine, dose of insulin glulisine, and total insulin dose were analysed using repeated measurement ANCOVA based on the ITT population. The geometric means for concentration data were calculated from the least squares means of logs for each treatment. The p values were based on the test of log ratios. The proportions of patients with weight increases of >5% or >10% baseline body weight and proportions achieving HbA_1c_ targets <7.0% and <6.5% were determined. Odds ratio (OR) estimates, 95% CI, and p values were established with logistic regression models including treatment arm, injection method, number of injections, metformin use, and baseline weight or HbA_1c_. The safety population was used for the analysis of hypoglycaemic event rates. The logistic regression model was used to assess the difference, if any, between the two treatment arms for symptomatic, severe, nocturnal, or serious hypoglycaemia. The analysis included the 95% CIs of ORs and p values. The number of episodes per patient-year was estimated for each event of hypoglycaemia by using the log-rank or Poisson regression model.

A trial with sample size of 105 evaluable subjects per treatment has 80% power to detect a treatment difference in body weight change of 1.5 kg in a superiority trial. With 105 evaluable subjects per arm, a non-inferiority hypothesis test with an equivalence limit of 1.5 kg also has 80% power. The standard deviation used in the power computations was *σ* = 3.83. This is the 95% upper confidence limit of the standard deviation of weight change from a previous insulin glulisine clinical trial.

## Results

### Patients

A total of 716 patients were screened for randomization; 371 patients did not meet criteria for study inclusion and 345 patients were randomized on a 1 : 1 basis to the premeal or postmeal insulin glulisine group. One randomized patient in the postmeal arm withdrew from the study before receiving the first dose of study medication and was therefore not included in the safety population. Thus, the safety population comprised 173 patients in the premeal group and 171 in the postmeal group. Overall, 229 patients (66%) completed the study; 32% of patients in the premeal group and 36% in the postmeal group discontinued the study for reasons including ‘did not wish to continue’ (16 and 17% of patients in the two groups, respectively), lost to follow-up (3.5 and 5.8%, respectively), adverse events (2.9, 4.1%), protocol violation (2.9, 1.2%), lack of efficacy [1 patient (0.6%) in the premeal group], death [2 patients (1.2%) in the premeal group], and other (4.6, 7.6%).

Patient demographics and baseline characteristics were similar for the two study arms (Table 3). Excluding the site with Good Clinical Practice non-compliance, the ITT set comprised 322 patients, with 163 patients in the premeal and 159 in the postmeal group. On average, the ITT group had a mean age of 54 years, approximately 56% were female, and BMI was 37 kg/m^2^. For the premeal and postmeal groups (ITT) baseline weight was 107 and 106 kg, HbA_1c_ level was 8.4 and 8.3%, and FPG level was 9.7 and 9.3 mmol/l (174 and 168 mg/dl), respectively. The PP population included 107 in the premeal group and 106 in the postmeal administration group. The mean body weight in the PP population at baseline was 106.5 kg (premeal) and 108.2 kg (postmeal). Demographics and background information were similar in all three populations studied.

**Table 3:** Patient baseline characteristics and demographics, safety population

	Premeal (n = 173)	Postmeal (n = 171)
Age, year (mean ± SD)	53.9 ± 9.2	53.7 ± 9.9
Female, n (%)	98 (56.6)	96 (56.1)
Race, n (%)		
White	129 (74.6)	125 (73.1)
Black	26 (15.0)	26 (15.2)
Asian	7 (4.0)	3 (1.8)
Multiracial	4 (2.3)	3 (1.8)
Other	7 (4.0)	14 (8.2)
Age at onset, year (mean ± SD)	41.2 ± 10.4	40.9 ± 9.7
Diabetes duration, year (mean ± SD)	13.9 ± 7.6	14.0 ± 6.9
Baseline HbA_1c_, % (mean ± SD)	8.4 ± 0.78	8.3 ± 0.75
Weight, kg (mean ± SD)	106.5 ± 24.1	105.4 ± 21.4
BMI, kg/m^2^(mean ± SD)	37.4 ± 8.0	37.0 ± 7.8
FPG, mmol/l (mean ± SD)	9.6 ± 3.6	9.5 ± 3.7
mg/dl (mean ± SD)	173 ± 65	170 ± 67
Number of injections, n (%)		
Two per day	42 (24.3)	47 (27.5)
More than two per day	131 (75.7)	124 (72.5)

BMI, body mass index; FPG, fasting plasma glucose; HbA_1c_, glycated haemoglobin A_1c_.

### Weight Change from Baseline to Study End

**Figure 1:**
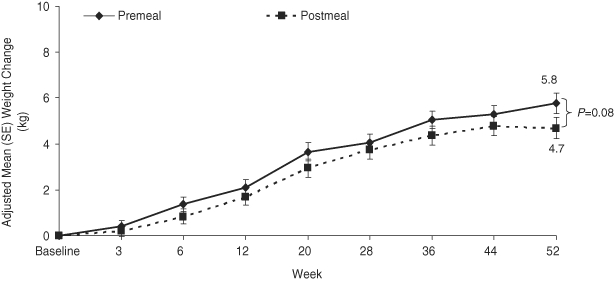
Adjusted mean (SE) change in premeal and postmeal weight from baseline to study endpoint (week 52) following insulin glulisine treatment across study weeks (visit weeks 0, 3, 6, 12, 20, 28, 36, 44 and 52). ITT, intent to treat.

Non-inferiority of weight change from baseline at the week 52 visit was shown. Adjusted means (SE) were 5.53 (0.61) kg and 4.66 (0.61) kg for the premeal and postmeal PP groups, respectively, and the upper 95% confidence bounds on the difference in mean weight change between the two treatment arms were within the non-inferiority margin of 1.5 kg specified in the study protocol [week 52: postmeal—premeal = −0.87 kg with 95% CI (–2.35 to 0.60)]. The difference in body weight change was not significant (p = NS). Similarly, when this comparison was repeated in the ITT population, the adjusted mean (SE) weight gain at study end was 5.42 (0.59) and 4.69 (0.60) kg (p = NS) in the premeal and postmeal treatment groups, respectively [week 52: postmeal—premeal = −0.73 kg with 95% CI (–2.17 to 0.71)]. The secondary, repeated measures analysis in the ITT population showed the mean weight gain across the 52 week time period (to weeks 3, 6, 12, 20, 28, 36, 44 and 52) was consistently numerically lower in the postmeal than in the premeal arm, although these differences were not significant at any time point (Figure 1) [week 52: postmeal—premeal = −1.1 kg with 95% CI (−2.28 to 0.13)]. Weight change across the 52-week time period was also examined for the PP population, with similar results [week 52: postmeal—premeal = −0.96 kg with 95% CI (−2.26 to 0.34)]. The proportion of patients with a weight increase of >5% or >10% of their body weight from baseline to endpoint did not reach statistical significance for premeal vs. postmeal administration [>5%: 45% of patients in the premeal group and 36% of patients in the postmeal group, OR (95% CI) = 0.68 (0.43, 1.06), p = 0.085; >10% : 14% and 10% of patients, OR (95% CI) = 0.71 (0.36, 1.14), p = 0.334; respectively].

### Insulin Dose

The adjusted mean titrated doses for insulins glulisine and glargine increased in both treatment arms over the course of the study with no difference (p ≥ 0.5) between treatment arms in insulin glulisine or glargine requirement. Patients in the premeal and postmeal arms achieved an adjusted mean total insulin dose of 2.37 and 2.23 U/kg, respectively, at study end (p = NS; ITT).

### Glycaemic Control

At study end, patients treated with basal insulin glargine plus premeal or postmeal insulin glulisine achieved similar reductions in adjusted mean FPG levels of 6.6 mmol/l (119 mg/dl) or 6.7 mmol/l (120 mg/dl), respectively. This represents a reduction of 2.1 mmol/l (38 mg/dl) for the premeal group and 2.0 mmol/l (37 mg/dl) for the postmeal group from baseline to endpoint (p = NS).

**Figure 2:**
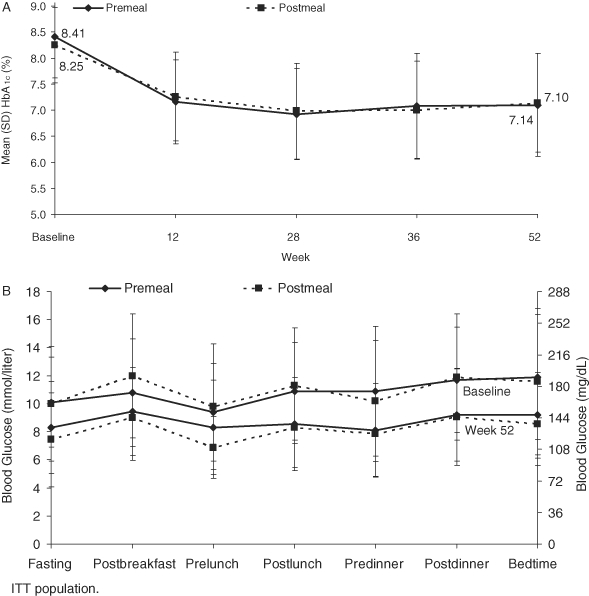
Effect of premeal and postmeal insulin glulisine treatment on HbA_1c_ levels. Panel A shows the change in mean (SD) HbA_1c_ across study weeks (visit weeks 0, 12, 28, 36 and 52). Panel B shows the seven-point blood glucose profiles for both treatment groups (showing mean (SD) values at each time point at baseline and at week 52). HbA_1c_, glycated hemoglobin A_1c_. ITT, intent to treat.

After 28 weeks, patients in the premeal and postmeal insulin glulisine group achieved similar reductions in HbA_1c_ (6.86 and 6.96%, respectively; p = NS). At week 52, patients treated with insulin glargine plus glulisine in the premeal arm achieved an HbA_1c_ level of 7.04 and 7.16% in postmeal arm. This represents a reduction of 1.28% for the premeal and 1.15% for the postmeal arm from baseline to endpoint (p = NS). HbA_1*c*_ measurements were comparable at visit weeks 12, 28, 36 and 52 for the two groups (Figure 2A). In addition, at study end, the percentages of patients with HbA_1c_ levels <7.0% and <6.5% were also not significantly different between treatment groups [<7.0%: 36% of patients in the premeal group and 33% of patients in the postmeal group, OR (95% CI) = 0.78 (0.49, 1.25), p = 0.305; <6.5%: 19 and 15% of patients, OR (95% CI) = 0.64 (0.35, 1.17), p = 0.147; respectively].

Changes in preprandial and postprandial BG levels from baseline at week 52 were captured by the seven-point BG profile, with a comparable decline in BG values observed in both treatment groups (Figure 2B).

### Hypoglycaemia

The incidence and rate of hypoglycaemic events were derived from the safety population (Table 4). The premeal treatment group had 128 severe episodes in 36 patients (20.8%) compared with 73 episodes in 30 patients (17.5%) in the postmeal group. Similarly, more patients in the premeal group (n = 8; 4.6%) experienced serious hypoglycaemia with 12 episodes compared with patients in the postmeal group (n = 3; 1.8%), who experienced 5 episodes. There was no significant difference in the rate of severe and serious hypoglycaemia between the premeal and postmeal treatment arms (0.89 vs. 0.54 events per patient-year; p = NS). However, compared with the postmeal cohort, patients in the premeal treatment arm experienced significantly lower rates of low [<2.8 mmol/l (<50 mg/dl)] and nocturnal (1.1 vs. 2.1 events per patient-year; p = 0.01) hypoglycaemic events, moderate and symptomatic [<3.9 mmol/l (<70 mg/dl); 29.0 vs. 37.0 events per patient-year; p = 0.05] hypoglycaemic events, and moderate and nocturnal (<3.9 mmol/l (<70 mg/dl); 4.5 vs. 7.7 events per patient-year; p = 0.0017] hypoglycaemic events (Table 4).

**Table 4:** Incidence and rate of hypoglycaemic events

Incidence	Premeal (n = 173)	Postmeal (n = 171)	p value
<3.9 mmol/l and symptomatic	146 (84.4)	143 (83.6)	0.8539
<3.9 mmol/l and nocturnal	113 (65.3)	119 (69.6)	0.3952
<2.8 mmol/l and symptomatic	104 (60.1)	106 (62.0)	0.8901
<2.8 mmol/l and nocturnal	54 (31.2)	66 (38.6)	0.1653
Severe hypoglycaemia and serious hypoglycaemia	36 (20.8)	30 (17.5)	0.4369

Events per patient-year ± SE	Premeal (n = 173)	Postmeal (n = 171)	p value
<3.9 mmol/l and symptomatic	29.0 ± 2.81	37.0 ± 3.55	0.05
<3.9 mmol/l and nocturnal	4.5 ± 0.60	7.7 ± 0.90	0.0017
<2.8 mmol/l and symptomatic	5.8 ± 0.85	7.5 ± 1.11	0.19
<2.8 mmol/l and nocturnal	1.1 ± 0.23	2.1 ± 0.42	0.01
Severe hypoglycaemia and serious hypoglycaemia	0.89 ± 0.23	0.5 ± 0.21	0.29

Severe hypoglycaemia was defined as an event necessitating the assistance of another party with either a recorded SMBG level of <2.0 mmol/l (<36 mg/dl) or an event requiring prompt response to treatment with oral carbohydrates, intravenous glucose or glucagon. A hypoglycaemic event was considered a serious adverse event if it was associated with coma/loss of consciousness or hypoglycaemia seizure/convulsion.

## Discussion

This randomized controlled study investigated the effect of postmeal insulin glulisine administration on weight gain and glycaemic control in patients with type 2 diabetes. The postprandial administration showed consistently less weight gain than preprandial administration throughout the 1-year study; however, this did not reach statistical significance at any time point. Fewer patients using postprandial administration compared with preprandial administration experienced a >5% increase in total body weight (trend). Effective glycaemic control with each respective basal-bolus regimen was maintained for the 12 month duration of the study in both groups. Both treatment groups achieved comparable reductions from baseline in HbA_1c_ level, with adjusted mean HbA_1c_ approaching 7.0% after 12 weeks of treatment. Hypoglycaemia incidence was similar between premeal and postmeal administration regimens, as was event rate of severe hypoglycaemia; however, the postmeal group had higher rates of low-to-moderate hypoglycaemic events, which reached statistical significance.

The pattern of overall weight change in this study is consistent with that seen in an earlier randomized controlled trial conducted by Bergenstal and colleagues that evaluated the effectiveness of using a simple algorithm to adjust mealtime insulin glulisine doses by weekly SMBG patterns vs. using insulin-to-carbohydrate ratios [14]. Over a treatment period of 24 weeks, patients utilizing the carbohydrate counting approach to adjust mealtime insulin glulisine experienced less weight gain compared with those using the simple algorithm (2.4 vs. 3.6 kg; p = 0.06 between-group difference at week 24). Common to both studies, the lower weight gain associated with postprandial insulin glulisine dosing or carbohydrate counting may likely be because of a relatively precise matching of prandial insulin to the actual food consumed, rather than needing to eat sufficient quantities to match insulin doses administered before a meal.

Taken together, the current results and those of Garg and colleagues suggest that, in patients with type 1 or type 2 diabetes, the use of insulin glulisine after the meal with dosing based on actual food consumption, may enable relatively precise matching of mealtime insulin needs, particularly for patients for whom food intake is difficult to anticipate and adjustment of meal size to insulin dose may not always be feasible.

Although preprandial dosing of RAI is considered to be the standard practice, postprandial administration of insulin glulisine has also been advocated by Mitri and colleagues [15], focusing on the potential benefit of postmeal administration of short-acting insulins for the weight management of type 2 diabetes. In addition, postprandial RAI dosing has already been successfully applied in a clinical practice model of intervention [16].

A limitation of the current study was that quality of life and treatment satisfaction measures were not obtained. Preprandial RAI administration requires adjusting the meal size to the insulin dose, whereas postprandial RAI administration allows adjustment of the dose to the actual meal consumed. Lack of dietary freedom has been shown to have a strong negative effect on quality of life [17], therefore, regimens that provide more flexibility may improve quality of life. Another limitation of this study is that no information was available pertaining to compliance of medication administration timing. As in usual practice, it is likely that some patients may not have rigorously followed the exact timing of insulin administration in relation to meals. However, this would have been the same for both groups, so it would not affect study outcomes. Future studies may want to explore the effects of the timing of insulin administration on quality of life and treatment satisfaction. Furthermore, generalizability to non-Caucasian populations may be limited, as this study population included a predominance of white patients and very few Asians, for example, as *β*-cell function and dietary patterns may differ between these patient groups.

Strengths of the current study include the use of both PP and ITT statistical analyses for non-inferiority tests. This provided the opportunity to assess weight change in both those with baseline and ≥1 post baseline assessment of weight at any time point with last observation carried forward (ITT analysis), and in a subpopulation of patients who had weight measurements at baseline and week 52 (PP analysis). The results were consistent across both statistical analyses populations, showing non-inferiority of weight change with the postprandial and preprandial RAI dosing regimens.

## Conclusion

This study shows the effectiveness and dosing flexibility of adding postprandial insulin glulisine to basal insulin glargine as well as overall feasibility of such approach in the management of type 2 diabetes when clinically indicated.
